# Practical Bias Correction in Aerial Surveys of Large Mammals: Validation of Hybrid Double-Observer with Sightability Method against Known Abundance of Feral Horse (*Equus caballus*) Populations

**DOI:** 10.1371/journal.pone.0154902

**Published:** 2016-05-03

**Authors:** Bruce C. Lubow, Jason I. Ransom

**Affiliations:** 1 Natural Resource Ecology Laboratory, Colorado State University, Fort Collins, Colorado, 80523, United States of America; 2 U.S. Geological Survey, Fort Collins Science Center, Fort Collins, Colorado, 80526, United States of America; University of Tasmania, AUSTRALIA

## Abstract

Reliably estimating wildlife abundance is fundamental to effective management. Aerial surveys are one of the only spatially robust tools for estimating large mammal populations, but statistical sampling methods are required to address detection biases that affect accuracy and precision of the estimates. Although various methods for correcting aerial survey bias are employed on large mammal species around the world, these have rarely been rigorously validated. Several populations of feral horses (*Equus caballus*) in the western United States have been intensively studied, resulting in identification of all unique individuals. This provided a rare opportunity to test aerial survey bias correction on populations of known abundance. We hypothesized that a hybrid method combining simultaneous double-observer and sightability bias correction techniques would accurately estimate abundance. We validated this integrated technique on populations of known size and also on a pair of surveys before and after a known number was removed. Our analysis identified several covariates across the surveys that explained and corrected biases in the estimates. All six tests on known populations produced estimates with deviations from the known value ranging from -8.5% to +13.7% and <0.7 standard errors. Precision varied widely, from 6.1% CV to 25.0% CV. In contrast, the pair of surveys conducted around a known management removal produced an estimated change in population between the surveys that was significantly larger than the known reduction. Although the deviation between was only 9.1%, the precision estimate (CV = 1.6%) may have been artificially low. It was apparent that use of a helicopter in those surveys perturbed the horses, introducing detection error and heterogeneity in a manner that could not be corrected by our statistical models. Our results validate the hybrid method, highlight its potentially broad applicability, identify some limitations, and provide insight and guidance for improving survey designs.

## Introduction

A cornerstone of effective wildlife conservation and management is the ability to estimate animal population sizes accurately and precisely. Complete counts without error are rarely possible due to imperfect sighting conditions and large geographic expanses that often characterize wildlife management areas. Aerial surveys are one of the only spatially robust tools for surveys of large mammal populations, but statistical sampling methods are required to address the many factors that introduce detection bias, and thus affect accuracy and precision of the estimates [1–3]. Up to one third or more of wild ungulates may be missed by uncorrected aerial counts [4–7], primarily due to heterogeneity of sighting conditions [8]. Some commonly used techniques for correcting raw counts do not adequately address these biases [9–12], and those that purport to do so have rarely been validated to confirm their accuracy, resulting in estimates with unknown residual bias. Reliable estimates must address precision as well as bias, with a tradeoff: more heterogeneous biases require more complex models to account for them, thereby reducing precision for a given amount of data.

Statistical sampling techniques have been used to estimate animal abundance for decades [10, 13]. One technique, a form of mark-resight known as the double-observer method, involves one observer recording the initial sighting (analogous to a mark) and a second observer either missing the same group or independently recording it (analogous to a resighting) [14]. This technique is based on the assumption that similar sighting probabilities for all animals or animal groups are experienced by a given observer. However, contrary to this assumption, sightability may be influenced by internal factors (e.g., aircraft type, observer fatigue, and observer skill), external factors (e.g., animal behavior, group size, and distance from observer), and environmental factors (e.g., cloud cover, sun angle, vegetation cover, and topography) [8, 15].

One alternative that addresses heterogeneity of sighting probability, the sightability bias correction technique (commonly known as the Idaho Model), handles inherent differences in sighting probability among animals by using a pre-calibrated model for correcting sightability bias [4]. This technique has been widely used for elk (*Cervus elaphus*) in North America and assumes that the initial model calibration, which typically relies on a radio-collared sample of the population, applies uniformly over space, time, and observers [16]. In other words, variance in some internal factors is left unexplained and variance in external and environmental factors may or may not be adequately explained across time and space as conditions deviate from those prevailing during the calibration phase.

Integrating some common population estimation techniques for wildlife can alleviate limitations of the individual methods and provide greater power and efficiency [9, 17–19]. One solution to the simultaneous double-observer assumption of uniform sighting probability is to combine it with a method similar to the sightability bias correction technique. Unlike traditional sightability bias correction, no prior calibration is done; instead, observers in the front and back seats of a survey aircraft independently either detect or fail to detect animal groups, while also recording covariate information for each observation. This allows sighting models for each observer under varying conditions to be developed from a single survey (or small number of similar surveys) without a prior calibration phase using radio collars or other artificial marks on the surveyed animals [20, 21]. While some covariates, such as group size, have been found to influence sighting probability across multiple surveys [8], it is important to note that such probabilities also may vary across surveys even when the same observers and same aircraft are employed [21]. This hybrid method is thus a potentially useful technique for estimating abundance of many large mammal populations under heterogeneous survey conditions.

Free-roaming horses (*Equus caballus*) present an acute management challenge across the world because their abundance can conflict with a variety of cultural, political, ecological, animal welfare, and land management considerations [22]. As such, they are frequently the focus of research aimed at estimating or reducing population size. Several populations of free-roaming feral horses in the American West offered a unique opportunity to test the accuracy of the hybrid double-observer sightability method by comparing aerial survey results to populations of independently known size. These populations were either isolated or enclosed and the subject of intensive research that resulted in every horse in each population being individually identified and cataloged. Thus, the true sizes of these closed populations were a known, established quantity at the time of our study.

Outside of these few specific populations of known size, estimating feral horse population size across the expansive prairies, scrublands, and deserts of the American West continues to be a daunting challenge for managers [23]. Modern survey methods based on double-observer statistical models have been applied in Australia for various taxa [24–27], but these studies could not validate the methodology with known population size. Because the double-observer methodology is inherently limited to only two independent sighting occasions (by front and back seat observers), concerns persist regarding the potential bias introduced by unmodeled heterogeneity in aerial surveys [1].

Our objective for this study was to combine the simultaneous double-observer and sightability bias correction techniques in an aerial application for estimating populations of free-roaming feral horses and validate the methodology against populations of known size. We determined the accuracy of this integrated technique by comparing our results against known population sizes, or with reference to pairs of surveys before and after a known number of horses was removed by management. Although validating the bias correcting ability of this method was our primary objective, we also evaluated the ability of our survey designs to achieving suitable precision in our abundance estimates and used these observations to provide guidance for future survey design. Although these tests were conducted on horses in the Western United States, we expect the results to apply more broadly to aerial surveys of other large terrestrial mammal species across a range of habitat types.

## Materials and Methods

### Ethics Statement

All data for this study were collected with the permission of the U.S. Bureau of Land Management (BLM) on public lands it administers, and involved BLM permitted aerial observations of feral horses protected under The Wild Free-Roaming Horses and Burros Act of 1971 (U.S. Public Law 91–195, as amended).

### Study Areas

**Fig 1:**
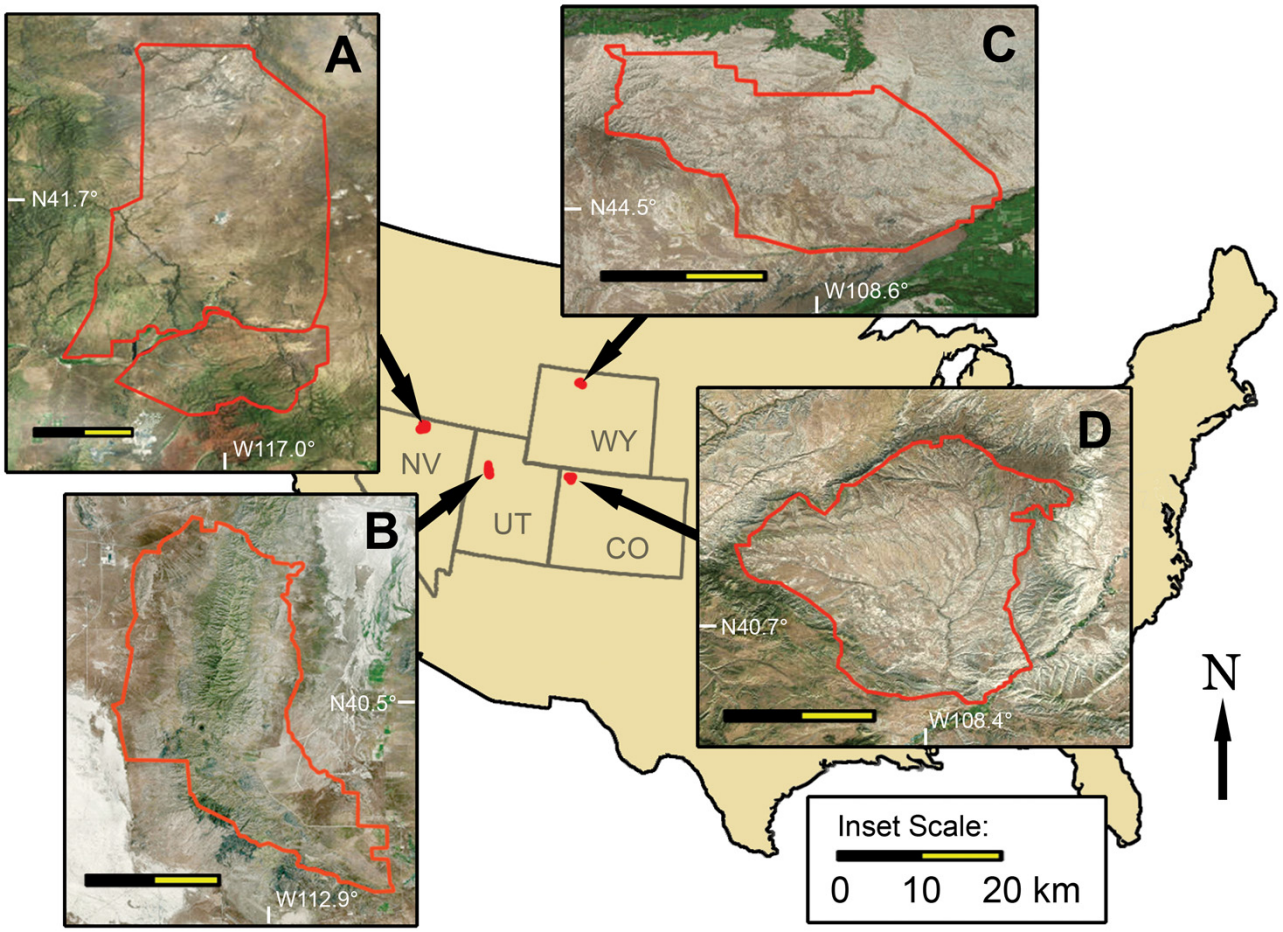
Map of aerial survey areas of feral horse (*Equus caballus*) populations in the United States. Location and topography of four U.S. Bureau of Land Management Herd Management Areas (HMA) surveyed: (A) Little Owyhee-Snowstorm Mountain HMAs, NV; (B) Cedar Mountain HMA, Utah; (C) McCullough Peaks HMA, Wyoming; and (D) Sand Wash HMA, Colorado, USA.

We conducted aerial surveys of feral horse populations in large areas of sage steppe habitat in the western United States in four Herd Management Areas (HMA) managed by the BLM: Cedar Mountain HMA, Utah (86,625 ha); contiguous Little Owyhee and Snowstorm Mountains HMAs, Nevada (233,490 ha); McCullough Peaks HMA, Wyoming (44,440 ha); and Sand Wash HMA, Colorado (63,390 ha) (Fig 1). These HMAs consisted of a mixture of flat, rolling, and rugged terrain populated predominately by sagebrush (*Artemisia* spp.), saltbush (*Atriplex* sp.), greasewood (*Sarcobatus* sp.), and various grasses. One area, Cedar Mountain, also included montane habitat where dominant tree species consisted primarily of piñon (*Pinus edulis*) and juniper (*Juniperus* sp.). Ungulates sympatric with feral horses in the study areas included livestock (*Bos* spp. and *Ovis aries*), elk (*Cervus elaphus*), mule deer (*Odocoileus hemionus*), and pronghorn (*Antilocapra americana*).

### Data Collection

We conducted three replicate tests of the hybrid double-observer technique at McCullough Peaks, two replicates at Sand Wash, and one at Cedar Mountain (S1 Table). We surveyed Little Owyhee-Snowstorm Mountains once before and again after a known management removal of horses. Transects at all four areas were predetermined and spaced systematically 1.6 km apart across each study area to provide complete coverage (rather than a geographic sample) of the entire area. Audio (radio silence) and visual (seat partition) isolation were maintained between front and back seat observers during the survey, with the provision that once a group of horses had passed the rear observer, all observers were free to discuss the count of group size, to record sightability covariate data, and to circle back if confirmation was needed. This procedure did not affect the sightability record, which was based solely on what observers detected while acting independently of one another.

Sightability covariates consisted of: time of day, observation direction, observer identity, seat position, number of animals, vegetation type (open, shrub, tree), approximate percent vegetation cover (nearest 10%), approximate percent snow cover (nearest 10%), approximate distance from aircraft (<0.4 km, 0.4–0.8 km, 0.9–1.6 km, and 1.6–3.2 km), terrain type (open, rugged), and behavior of horses (still or moving). Photographs were also taken by the front seat observer of all groups comprised of ≥10 individuals in order to ensure the correct count. Data were collected without replacement; in other words, groups were only counted once even if observed again. Group size and age composition along with individual markings were used to recognize groups that had already been recorded but had moved to a previously unsurveyed part of the study area where they were seen again. Photographs were used to verify the identity of groups if the group was moving and observers were uncertain of their identity.

The McCullough Peaks area was closely monitored on a weekly basis as part of a fertility control study and every individual horse in the population was known, enumerated, and catalogued [28]. Horses, unlike most North American mammals, each have unique pelage markings and characteristics that allow identification of individuals. The true population was therefore known with near certainty, excepting eight individuals that could not be located within the survey area and verified as alive on the day of the surveys. We surveyed this area using a Cessna^™^ 210 airplane with three observers. Two observers used the simultaneous double-observer method on the right side of the aircraft and the third observer was seated behind the pilot, collecting data singularly from the left. Rear observers switched sides at every refueling stop to allow each rear observer to be paired with the front observer periodically throughout the survey and in similar sighting conditions. The aircraft maintained an above-ground altitude of approximately 150–180 m and airspeed of approximately 220–260 km/h. Survey 1 transects were oriented east-west, Survey 2 were north-south, and Survey 3 were northwest-southeast. Flight paths and group locations were recorded using a Garmin 76S Map handheld global positioning system (GPS) unit with an external antenna mounted in the front window. Two additional surveys using this same integrated technique and protocol, in the same airplane, were conducted at Sand Wash. Survey 1 was flown with transects oriented north-south and Survey 2 was flown with transects oriented east-west. This study area was also closely monitored on a weekly basis as part of a fertility control study and every individual horse in the population was known by its unique pelage markings, enumerated, and catalogued (unpublished data, Humane Society of the United States).

Aerial surveys were conducted from a Bell 206BIII Jet Ranger^™^ helicopter at Little Owyhee-Snowstorm Mountains before and after a known management removal. Individual identity of horses was not known at this location, but the exact number of animals removed between surveys by management was known and no births and negligible deaths were expected in the intervening interval. All transects were flown east-west. The helicopter maintained an above-ground altitude of approximately 60–70 m and airspeed of 145–165 km/h. One front observer and one rear observer used the hybrid double-observer method on every other transect, with the rear observer collecting simultaneous data on alternating transects and singular data in between (this observer was always looking north).

A similar helicopter survey was conducted at Cedar Mountain using a Bell 206L Long Ranger^™^, and with the same methodology as the Little Owyhee-Snowstorm Mountains surveys. The larger helicopter at Cedar Mountain was employed to accommodate two rear observers and the montane conditions. Like Sand Wash, Cedar Mountain horses were also part of a fertility control project where every individual horse in the population was known by its unique pelage markings, enumerated, and catalogued (unpublished data, Humane Society of the United States).

### Data Analyses

We used the Huggins [29, 30] closed capture estimator for mark-resight data with individual covariates to estimate abundance. The model was structured with two capture occasions corresponding to the combined front observers (or single observer when the pilot did not participate) and the combined back seat observers (or single back seat observer). The units of measure in the mark-resight model were horse groups and not individual horses, because only groups were sighted independently. Sighting probabilities unique to each horse group and observer position were estimated by regression of a logistic model following Griffin et al. [20, 31], but using a different list of covariates as predictors of sighting probability.

We constructed separate sets of models for each of the four study areas with parameters selected from among 18 candidates (in addition to an intercept) believed to have the potential to influence sighting probability, although none of our final models included more than ten parameters (Table 1). We modeled front seat and back seat sighting probabilities with different models, but structured them with the same parameter values for all covariates except those specific to the seat position: *P*_*p*_, *P*_*b*_, *P*_*c*_, *O*_*b*_, *O*_*1*_, and *O*_*2*_ (Table 1). We modeled effects of back seat position in one of three ways: either a single average effect for back seat position (*O*_*b*_), two separate effects for each back seat observer (*O*_*1*_ and *O*_*2*_), or no effect for the back seat (i.e., no difference between front and back seat observers). All other variables had two possibilities: included or excluded in a given alternative model. Surveys used either helicopters or airplanes, but these aircraft types were confounded with survey location, so we could not model differences in sighting probability due to aircraft type separately from differences among study areas.

**Table 1:** Definitions of covariates considered in sighting probability models for feral horses (*Equus caballus*) in western USA.

Covariate	Description
*P*_*c*_	*P*_*c*_ = 1 if the horse group position was in the center, directly under the aircraft and not available to back seat observers.
*P*_*b*_	*P*_*b*_ = 1 if the horse group was spread widely across both sides of the flight path and available to all 4 observers.
*P*_*p*_	*P*_*p*_ = 1 if the horse group position was on the pilot’s side of the aircraft. This effect was only used to model front seat observer sighting probability.
*T*_*2*_	*T*_*2*_ = 1 for second (November) survey at Little Owyhee-Snowstorm Mountains.
*G*	Group Size: the number of individual horses in the group.
*A*	*A* = 1 if the horse group was moving when first sighted.
*R*	*R* = 1 if the horse group was located in rugged topography.
*C*_*o*_	*C*_*o*_ = 1 if the horse group was located in the open with no obstructing vegetation (not shrub or tree cover).
*C*_*t*_	*C*_*t*_ = 1 if the horse group was located in an area vegetated with trees (not open or shrub cover).
*V*	The percentage (in increments of 10%) of potentially obstructing vegetation (either shrub or tree) in the area where horses were first sighted.
*S*	The percentage (in increments of 10%) of snow cover in the area where horses were first sighted.
*S*_*2*_	The percentage of snow cover squared (*S*_*2*_ = *S*^2^).
*D*	Shortest distance from the predefined transect to the location where horses were first sighted, recorded as one of 4 categories: 0–0.4, 0.4–0.8, 0.8–1.6, and 1.6–3.2 km. These categories are represented in the analysis by their midpoints.
*L*_*p*_	*L*_*p*_ = 1 if lighting was partial sun and shadows (not overcast or full sun).
*L*_*o*_	*L*_*o*_ = 1 if lighting was over cast (not partial or full sun).
*O*_*b*_	*O*_*b*_ = 1 for predicting back seat observer’s sighting probability.
*O*_*1*_	*O*_*1*_ = 1 if the group was on the side of the aircraft visible to back seat observer 1.
*O*_*2*_	*O*_*2*_ = 1 if the group was on the side of the aircraft visible to back seat observer 2.

We did not consider categorical effects for which there were ≤5 observations for either value (presence or absence of the condition). For quantitative effects (*V*, *S*, and *D*) we did not include parameters unless there were >5 observations by both front and back observers for ≥2 values (Table 1). We only collected data for *P*_*c*_ at Cedar Mountain and for *P*_*b*_ at Little Owyhee-Snowstorm Mountains, and included parameters for these effects in the corresponding models. An additive effect for occasion, *T*_*2*_, was considered at Little Owyhee-Snowstorm Mountains where the two surveys were conducted three months apart in different seasons (autumn and winter). We also decided *a priori* that only one sign on certain parameters would be considered (positive for *G* and *C*_*O*_; negative for *P*_*p*_, *R*, *C*_*t*_, *V*, and *D*). These assumptions were enforced by constraining the model fitting. We made no *a priori* assumptions about the signs of the remaining parameters. We chose to include both a linear and quadratic effect of snow cover to model the likely possibility that partial, patchy snow cover could be detrimental to sighting probability. When data were sufficient to include a snow effect we always included both terms or neither, but never one without the other.

We fit a balanced set of alternative models with all possible combinations of the parameters we chose to test for support. Our final population estimates are based on weighted averages across all of these models based on AIC_*c*_ model weights [32]. Confidence intervals and other measures of precision (SE, CV) are based on 100 estimates: the original and 99 bootstrap runs [20, 31].

## Results

**Fig 2:**
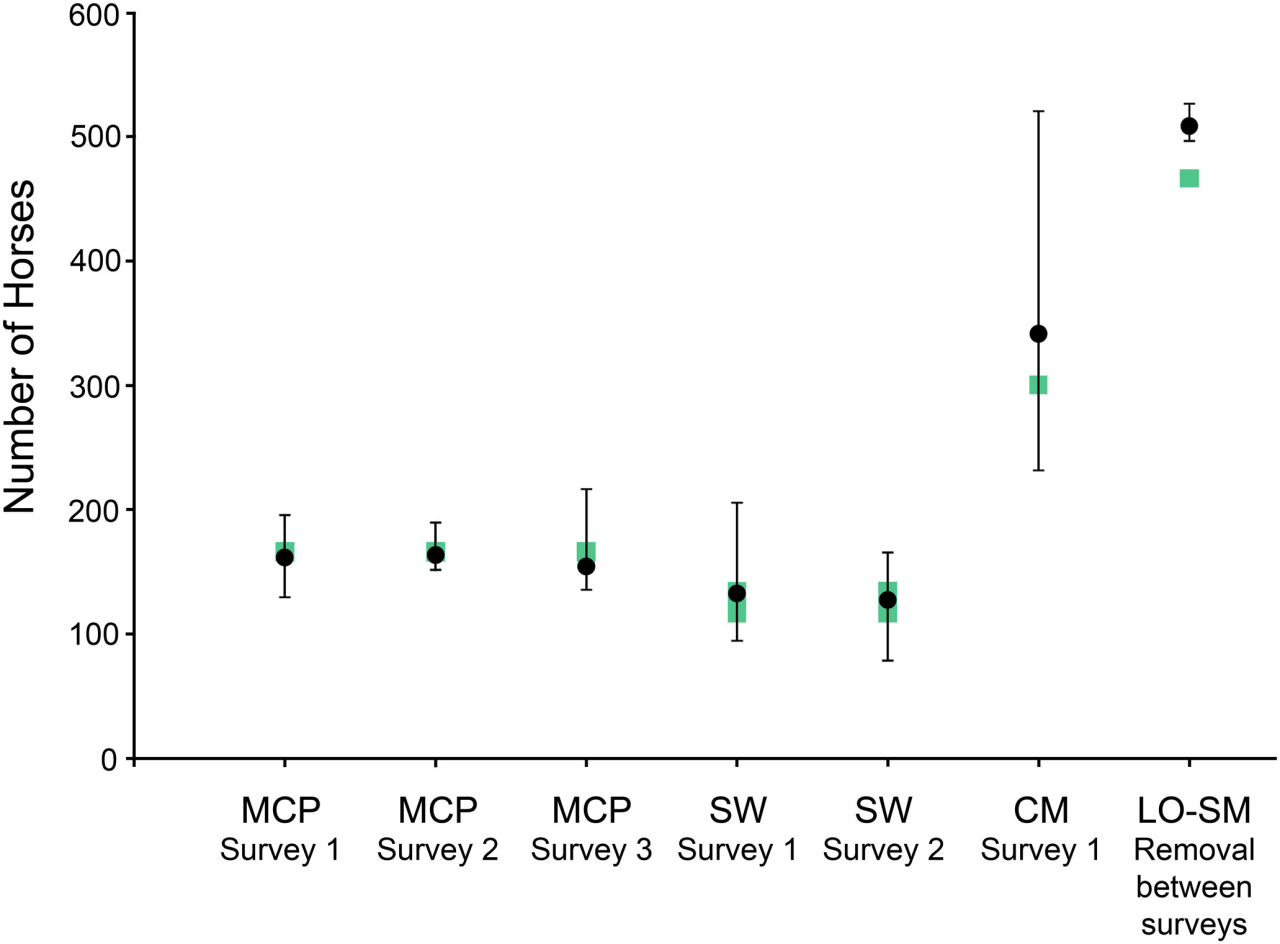
Comparison of estimates to known abundance for aerial surveys of feral horse (*Equus caballus*) populations. Abundance estimates or change in abundance (black dots) and 95% confidence intervals for aerial surveys of feral horse (*Equus caballus*) populations in the western USA, using a hybrid double-observer method. Ranges of known population sizes are shown in green. Surveyed U.S. Bureau of Land Management Herd Management Areas (HMA): McCullough Peaks HMA, Wyoming (MCP); Sand Wash HMA, Colorado (SW); Cedar Mountain HMA, Utah (CM); and Little Owyhee-Snowstorm Mountains HMAs, Nevada (LO-SM).

The three surveys at McCullough Peaks, two at Sand Wash, and one at Cedar Mountain all produced estimates that were close to the known population size with deviations from the known value (or the mean of the range of known values) ranging from -8.5% to +13.7% (Table 2, Fig 2). All of these estimates were ≤0.7 standard errors from the known population size and the 95% confidence intervals encompassed the known values in all cases. Precision of the estimates varied widely, from as low as 6.1% CV on one airplane survey at McCullough Peaks, up to 25.0% in the helicopter survey at Cedar Mountain. Lower precision (higher CV) corresponded to lower sighting probabilities: the estimated portion of the population missed by all observers ranged from 3.0% to 51.5% (Table 2).

**Table 2:** Abundance or removal estimates from aerial surveys and known population or removal sizes of feral horses (*Equus caballus)* in western USA. U.S. Bureau of Land Management Herd Management Area (HMA) surveys: McCullough HMA, Wyoming (MCP), 3 replicates; Sand Wash HMA, Colorado (SW), two replicates; Cedar Mountain HMA, Utah (CM); and change due to removal at Little Owyhee-Snowstorm Mountains HMAs, Nevada (LO-SM), USA. Aircraft types: airplane (AP) and helicopter (HELO).

	Survey						
HMA	MCP			SW		CM	LO-SM
Survey replicate	1	2	3	1	2		
Month(s)	Feb	Feb	Feb	Feb	Feb	Feb	Aug, Nov
Aircraft type	AP	AP	AP	AP	AP	HELO	HELO
Known min pop	166	166	166	117	117	301	n/a
Known max pop	174	174	174	142	142	301	n/a
Known removal	n/a	n/a	n/a	n/a	n/a	n/a	467
Estimate	162	164	155	133	128^a^	342	509
95% LCL ^b^	130	152	136	95	79	232	497
95% UCL ^b^	196	190	217	206	166	521	527
Standard Error	14.9	9.9	23	31.6	24.4	85.7	8
Coefficient of Variation	9.2%	6.1%	14.9%	23.7%	22.5%	25.0%	1.6%
No. of horse groups seen	16	20	21	21	14	44	104
No. Horses Seen	157	159	146	87	66	166	507
% Missed	3.0%	3.1%	5.8%	34.7%	39.0%	51.5%	0.5%
Deviation ^c^	-8	-6	-15	4	-1	41	42
Deviation (%) ^c^	-4.8%	-3.5%	-8.8%	2.8%	-1.0%	13.7%	9.1%
Deviation/SE ^c^	-0.5	-0.6	-0.7	0.1	-0.1	0.5	5.3

^a^ Added 20 horses from outside HMA estimated from first replicate, not surveyed again on second replicate.

^b^ Lower and upper bounds are for 95% confidence intervals based on bootstrap results.

^c^ The deviation is the estimated number of horses minus the known value or the mean of the minimum and maximum known values when these differ. The deviation% is the deviation divided by the known value or the mean of the minimum and maximum known values. The deviation/SE is the deviation divided by the standard error.

The pair of surveys conducted at Little Owyhee-Snowstorm Mountains produced an estimated change in population between the surveys that was significantly larger than the known decline resulting from the management removal. This could result from overestimating the initial population, underestimating the final population, or a combination of both. The estimated precision and overall sighting probabilities were extraordinarily high for the individual estimates before and after the removal and, consequently, for the estimated removal. Although the error between the estimated number of animals removed (509) and known number of animals removed (467) was a modest 9.1%, the high precision of the estimate meant that the difference represented 5.3 standard errors and that the 95% confidence interval did not include the known value (Table 2).

The cumulative number of observed horse groups ranged from 35 at Sand Wash to 104 at Little Owyhee-Snowstorm Mountains (where there were multiple observations of the same groups over the course of multiple survey replicates at these locations). Observed group size varied widely from median = 3, mean = 3.8, and maximum = 9 horses at Cedar Mountain to median = 6, mean = 8.6, and maximum = 84 horses at Little Owyhee-Snowstorm Mountains. The distribution of group size was highly skewed for some areas, so we chose to use the natural logarithm transformation of this covariate in those cases where the mean/median ratio was >1.3 (McCullough Peaks, 2.0, and Little Owyhee-Snowstorm Mountains, 1.4) to obtain a more normally distributed covariate. Preliminary analyses confirmed that this transformation improved AIC_*c*_ model weight.

We determined during preliminary analyses that *P*_*p*_ was overwhelmingly supported (>99% AIC_*c*_ model weight) for all study areas except Little Owyhee-Snowstorm Mountains and chose to include this parameter in all models for those areas in the final model set. We also found overwhelming support for *P*_*c*_, and *P*_*b*_ in the locations where these data were recorded (Cedar Mountain and Little Owyhee-Snowstorm Mountains, respectively) and included these effects in all models in our final model set for those areas. After eliminating candidate parameters that did not meet our sample size criteria (Table 3), the maximum number of parameters considered for McCullough Peaks was eight, with two included in all models and up to two for the back seat effects resulting in 2^4^ * 3 = 48 models. For Sand Wash, we considered ≤10 parameters, two in all models and up to two for the back seat, leaving six to be included or excluded; however, we always included or excluded the pair of snow effects (*S* and *S*_*2*_) together, so there were 2^5^ * 3 = 96 models. Cedar Mountain models had ≤9 parameters, three included in all models and up to two for back seat observers resulting in 2^4^ * 3 = 48 models. Little Owyhee-Snowstorm Mountains had ≤7 parameters, with two always included. Only one back seat observer was present at Little Owyhee-Snowstorm Mountains, so the back seat was modeled with either one or zero parameters resulting in 2^5^ = 32 models.

**Table 3:** Sample size, AIC_*c*_ model support, and parameter estimates for models of feral horse (*Equus caballus*) abundance. U.S. Bureau of Land Management Herd Management Areas (HMA) Surveyed: McCullough Peaks HMA, Wyoming (MCP), Sand Wash HMA, Colorado (SW), Cedar Mountain HMA, Utah (CM), and removal at Little Owyhee-Snowstorm Mountains HMAs, Nevada (LO-SM), USA. Sample unit is horse groups, not individual horses.

	Sample Size: n / (N—n)				AIC_*c*_ Model Weight^a^				Estimated Parameter Value^b^			
	MCP	SW	CM	LO-SM	MCP	SW	CM	LO-SM	MCP	SW	CM	LO-SM
Int (n)	57	35	44	104	100%	100%	100%	100%	--	--	--	--
Center (*P*_*c*_)	0/57	0 / 35	7 / 37	--	*	*	100%	--	*	*	-2.428/-6.993	--
Both (*P*_*b*_)	--	--	--	9/95	--	--	--	100%	--	--	--	1.76/5.92
Survey (*T*_*2*_)	--	--	--	37/67	--	--	--	36%	--	--	--	-0.314/-1.39
Pilot Side (*P*_*p*_)	31 / 26	12 / 23	15 / 29	49/55	100%	100%	100%	99%	-19.0/-38.8	-3.90/-8.81	-2.023/-4.93	-6.36/-13.62
Group Size^c^ (*G*)	57 / 0	35 / 0	44 / 0	104/0	89.0%	20.9%	**	92%	1.56/-0.063	0.008/-1.94	**	1.22/-0.588
Activity (*A*)	3 / 54	4 / 31	29 / 15	0/104	*	*	25%	*	*	*	0.155/-1.05	*
Rugged (*R*)	0 / 57	2 / 33	29 / 15	1/103	*	*	50%	*	*	*	-0.712/-2.86	*
Open (*C*_*o*_)	18 / 39	2 / 33	15 / 29	0/104	24.7%	*	**	*	0.139/-0.191	*	**	*
Tree (*C*_*t*_)	0 / 57	0 / 35	16 / 28	0/104	*	*	35%	*	*	*	-0.460/-1.69	*
Veg%^d^ (*V*)	39 / 18	33 / 2	30 / 14	0/104	24.8%	20.9%	*	*	-0.040/-1.26	0.020/-2.21	*	*
Snow^d^ (*S*)	0 / 57	21 / 14	16 / 28	0/104	*	12.2%	*	*	*	-0.690/-2.77	*	*
Snow^d^ (*S*_*2*_)	0 / 57	21 / 14	16 / 28	0/104	*	12.2%	*	*	*	0.767/1.53	*	*
Distance^e^ (*D*)	25 / 23	16 / 19	20 / 24	79/25	23.6%	22.0%	52%	100%	0.014/-1.176	-0.240/-3.23	-1.24/-7.07	-5.83/-14.3
Light: partial (*L*_*p*_)	0 / 57	26 / 9	5 / 39	--	*	23.5%	*	--	*	-0.136/-1.98	*	--
Light: overcast (*L*_*o*_)	0 / 57	5 / 30	39 / 5	--	*	*	*	--	*	*	*	--
Back (*O*_*b*_)	54	26	29	95	21.9%	25.5%	27%	37%	--	--	--	--
Back Obs 1 (*O*_*1*_)	24 / 33	20 / 15	17 / 27	104/0	6.4%	34.8%	11%	*	-0.004/-0.852	-0.062/-1.26	0.003/-0.779	*
Back Obs 2 (*O*_*2*_)	33 / 24	15 / 20	20 / 24	--	6.4%	34.8%	11%	*	0.006/-1.15	-0.644/-2.14	-0.092/-1.11	*

^a^ Sum of AICc model weights for all of the alternative models that included a given parameter. Each parameter can have 0–100% support. Weights do not add to 100% across all parameters because parameters are included in multiple models. Cases where model weight is 100% represent parameters that were included in every alternative model by *a priori* decision.

^b^ Parameter calculated from raw covariate values / parameter calculated from values standardized by subtracting the mean and dividing by the standard deviation of all covariate values observed at each given study area.

^c^ Natural logarithm of group size used for MCP and LO.

^d^ Number of values >0 / = 0

^e^ Number of values = 0–0.4 km / >0–0.4 km.

^(--)^ Not applicable or data not collected

* Parameter not considered due to insufficient data or insufficient variation in values.

** Parameter excluded from final model set based on preliminary analysis indicating lack of support and implausible sign.

At McCullough Peaks, 57 horse groups containing 462 individual horses were observed cumulatively over the course of the three surveys. Of the effects tested, there was strong support for only the ln(*G*) effect (Table 3). Support for the remaining effects tested was weak (<30% AIC_*c*_ model weight summed across models containing each parameter). Observers recorded 35 horse groups and 153 horses during the two surveys at Sand Wash. Support was weak (<39%) for all of the effects considered in candidate models, other than for the individual back seat observers, which received only modest support. The 44 groups seen at Cedar Mountain contained 166 horses. There was moderate support for the *D*, *T*, and *R* effects, which all reduced sighting probability as expected. During the two surveys at Little Owyhee-Snowstorm Mountains, 104 horse groups containing 895 horses were observed. Very uniform conditions at Little Owyhee-Snowstorm Mountains provided minimal data for several covariates; nevertheless, there was very strong support (>90%) for *P*_*p*_, ln(*G*) and *D* and modest support for *O*_*b*_. Recall that in addition to the effects tested, all models included an intercept. Models for all areas except Little Owyhee-Snowstorm Mountains included *P*_*p*_, the model for Cedar Mountain included *P*_*c*_, and the model for Little Owyhee-Snowstorm Mountains included *P*_*b*_ based on overwhelming support (>99%) for these effects.

## Discussion

In all six tests where the population was known independently with a high degree of confidence, our estimates based on double-observers and modeling of covariate effects on sighting probability produced excellent estimates with confidence intervals that easily encompassed the known values. It is reassuring that the estimates appear to be unbiased even when sighting probability was low and large corrections for unobserved horses were necessary. Avoiding bias to achieve accuracy objectives can be difficult because it requires developing reliable models of sighting probability that account for heterogeneous biases for each animal group. To accomplish this, the covariates most likely to explain a substantial portion of this heterogeneity must be identified, recorded, and statistically examined for their relative importance. Our candidate covariate list and sample sizes for our surveys appeared to be adequate to model sighting heterogeneity sufficiently to correct obvious biases; however, we caution that larger sample sizes would be required to adequately estimate more complex models when more heterogeneous sighting conditions prevailed. Unfortunately, the success of these models in correcting biases in abundance estimates cannot be determined from the data themselves, but only by comparison to an independent source of unbiased values, as we have done here.

Despite the apparent success in achieving unbiased estimates in our six surveys of known populations, only the three surveys at McCullough Peaks can be judged a full success, because the precision of the Sand Wash and Cedar Mountain surveys are likely too low for most management purposes. Low precision in those areas was largely a consequence of low sighting probabilities and small sample sizes. Achieving a desired level of precision in aerial surveys is primarily a function of sample size and underlying variability. In abundance estimation, sample size is a function of the number of animal groups (not individual animals) present and the proportion of them that are seen. Variability is largely a function of sighting probability and heterogeneity of sighting probability among groups: low sighting probability requires large statistical corrections with correspondingly large contribution to the estimated error and more heterogeneity requires more data to estimate more model parameters.

The population surveyed can be increased by pooling either spatially by surveying more management units, or temporally by repeating surveys. In areas where several adjacent or nearby populations exist, it is good practice to survey these entire ‘complexes’ together to properly address geographic closure. This practice provides the added benefit of larger sample sizes, which contribute more robust information toward the overall population estimate [33]. Pooling data may also be justified across multiple surveys of the same area. For either spatial or temporal pooling to be successful, it is important to hold as many variables constant as possible, including: aircraft type, pilot and observers, season, weather, transect spacing, aircraft speed and altitude.

Variability can be reduced while simultaneously increasing sample size by increasing sighting probability. Survey design can influence sighting probability through the choice of: survey time that optimizes environmental factors such as season and weather conditions; internal factors such as the aircraft type, and the number, skill, and experience of observers used; and, most importantly, survey effort. More intensive (and therefore expensive) surveys with more tightly spaced transects flown at slower speeds and lower altitude can increase sighting probability. Thus, an important lesson from our results is that transects must be spaced sufficiently close together, flown at a slow enough speed, and low enough altitude to ensure high sighting probabilities—that is, >90% for the combined observers, which is typically possible with >70% sighting probability for each observer position (front and back). For any given study area, a pilot survey or repeated surveys may be required to determine the level of survey effort needed to obtain sufficient sighting probability and sample size to achieve the precision required.

The pair of surveys at Little Owyhee-Snowstorm Mountains produced an estimate of the intervening population removal that did not agree with the actual removal. It is noteworthy that the estimate of the number removed was biased high by only 9.1%. Far more concerning was the very high estimated precision that made this modest absolute error appear to be highly statistically significant. It may be that the point estimate was not badly flawed, but that the variance estimate was. The observers reported that nearly every horse group was disturbed by the approaching helicopter and responded by running long before the helicopter reached it. This made tracking horse groups difficult. The problem was especially acute during the first survey because the larger, denser population resulted in a ‘domino effect’ of groups running in response to other groups running. We believe that this behavioral response resulted in two effects that contributed to the overly-precise results. It is likely that some groups escaped detection because they ran into areas that had already been surveyed. In contrast, other running groups were easily spotted by both observers because of the increased visibility of a moving object, especially when accompanied by a dust trail that is visible from a substantial distance. It is possible that some groups were mistakenly seen and counted twice (as was reported by Linklater and Cameron [34]). Such a pattern would be consistent with many highly visible groups detected by both observers and a few groups essentially not visible because they were never present at the same time and place as the helicopter. These circumstances explain the apparent (but likely misleading) high estimated sighting probability and precision, and apparent bias in the estimate. When both observers tend to either see or miss groups due to factors that are not modeled, the result is unmodeled heterogeneity and an erroneously high estimate of detection probability [20, 35]. This problem may have been exacerbated by the fact that this was the only survey where only one observer was positioned in the back seat. Finally, estimating a change in population abundance requires two surveys, and problems with either one would be sufficient to create erroneous estimates—it is not clear whether one or both surveys of this area were flawed, or by how much.

We found overwhelming support for all three of the effects related to horse group position: *P*_*p*_, *P*_*c*_, and *P*_*b*_. It was entirely expected that sighting probability for the front seat would be lower on the pilot’s side of any aircraft, given the pilot’s primary responsibility was to navigate and fly safely. We would have included indicators for center and both sides in all surveys had we recognized their importance earlier. Collecting data on all three of these positions may improve models of sighting probability. We also obtained moderate to strong support from ≥1 survey area for effects of group size, *G*, rugged topography, *R*, and back seat observer position, *O*_*b*_. The lack of strong support for the remaining effects are likely due to two factors: 1) small total sample size (number of observed groups) and 2) relatively uniform conditions at many of these study areas resulting in even smaller effective sample size for a given condition. The absence of strong support for these effects does not rule them out as potentially valuable for modeling heterogeneity in sighting probabilities for studies with larger sample sizes and in more complex study areas. The likely importance of these additional covariates warrants consideration in planning future surveys and inclusion if local observers with aerial survey experience suspect they are related to sighting probability at a given location. In a more general look at detection bias in aerial surveys of feral horses, it has also been noted that in addition to group size and the covariates we considered, observer fatigue and sun effect may also play a role in some situations [8]. Mosaics of snow and vegetation, horse pelage patterns, and interactions of those patterns with sun, may further complicate detection probability, and such measures used as covariates may only be helpful if sample sizes are sufficiently large and conditions are heterogeneous enough to be statistically informative about the effects of those covariates on observers’ detection probabilities.

We caution that all of the study areas included in this test were relatively open, flat, and sparsely vegetated, except Cedar Mountain, which included both open sage steppe and more heavily vegetated montane habitats. In areas with high topographic and vegetation variability, and thus more obstructions to visibility, we expect stronger support for more of the measured effects on sighting probability. However, more heterogeneous sighting conditions can greatly increase uncertainty around the estimate and would require a larger sample size to obtain the same level of precision. Given the small sample size, low sighting probability, and greater heterogeneity at Cedar Mountain, it is not surprising that estimates there exhibited the lowest precision of all the surveys. In even more extreme cases where sighting probabilities can vary widely among groups due to factors that cannot be fully accounted for by a handful of sighting covariates, the method presented here is unlikely to be accurate. Where there is extreme heterogeneity of sighting probabilities and uncertainty about the causes of that heterogeneity, we previously demonstrated that a mark-resighting survey using photographs to identify natural markings is effective [9]. In that study, we noted that using only two sighting occasions in such heterogeneous sighting conditions would have led to estimates as much as 22.7% under the known population size. For species without natural markings, artificial markings (i.e. radio collars) can be used to model heterogeneity that cannot be explicitly accounted for using a small set of measurable covariates [20].

## Conclusion

Statistical methods can correct inherent biases in aerial surveys, but it is vital that the underlying assumptions of the statistical methods are satisfied. No analysis can overcome inherent limitations in a poorly designed or executed survey. Improvement in precision can be obtained by heeding the lessons learned in this study and designing surveys to ensure sufficient sighting probability and sample size. The problems at Little Owyhee-Snowstorm Mountains suggest that airplanes are preferable to helicopters whenever terrain does not require the use of a helicopter. In many areas, helicopters cause behavioral reaction by animals, which can complicate detection and sighting probability. Statistical sampling techniques cannot adequately correct for this source of bias, where the animals being surveyed react to the observer in ways that systematically inflate or depress detection. It is also vital that two observers be positioned in the back seat and one observer in the front, in addition to the pilot, to maximize observation opportunities. Although we did not explicitly investigate this issue, we believe that photographing large animal groups, as we did in this study, is also essential for accurate determination of group size to avoid another source of negative bias, as has been shown previously [21, 36]. Careful survey planning and design including the extent of the survey (for adequate sample size and geographic closure), skill and number of observers, the type, flight speed and altitude of the aircraft, the spacing of transects, the choice of covariate data collected, season and weather conditions, all contribute to the ultimate success of a survey in terms of both accuracy and precision.

Accurately estimating large mammal abundance is an on-going challenge with increasingly more specific management objectives [23]. The acceptable levels of precision and accuracy in aerial surveys can only be determined by the management need [37]. There may be more than one methodological solution for these needs for a wide range of environments with vastly different visual characteristics, geographic size, and animal abundance. A growing suite of abundance estimating tools has now become available to managers and use of statistically valid sampling techniques is essential for arriving at wildlife population estimates with quantified bounds. Previous applications of the hybrid simultaneous double-observer method that incorporate sightability bias correction with covariates, presented here, to elk in heavily vegetated and mountainous [20] and arid habitats [21] and now to feral horses in open sage steppe, desert, semi-arid and arid grassland, and montane habitats suggest that this is a potentially valuable tool for estimating abundance of numerous large terrestrial mammals species in a variety of habitats around the world.

## Supporting Information

S1 Table Data collected during aerial surveys of feral horses (*Equus caballus*) at U.S. Bureau of Land Management Herd Management Areas (HMA): McCullough Peaks HMA, Wyoming; Sand Wash HMA, Colorado; Cedar Mountain HMA, Utah; and Little Owyhee-Snowstorm Mountains HMAs, Nevada. (CSV)
